# Effectiveness of vestibular physical therapy on balance and dizziness in people with multiple sclerosis: A systematic review and meta-analysis

**DOI:** 10.1177/09574271261444952

**Published:** 2026-05-05

**Authors:** Diego Piatti, Gianluca Paolocci, Daniel Hector Verdecchia, Maria Grazia Grasso, Gianfranco Bosco, Iole Indovina, Marco Tramontano

**Affiliations:** 1 Laboratory of Neuromotor Physiology, IRCCS Santa Lucia Foundation, Rome, Italy; 2 Department of Medical, Surgical and Technological Sciences, University of Catania, Catania, Italy; 3 Department of Health Sciences, 28224 Universidad Nacional de La Matanza, Buenos Aires, Argentina; 4 Multiple Sclerosis Unit, IRCCS Santa Lucia Foundation, Rome, Italy; 5 Department of Systems Medicine and Centre for Space BioMedicine, 9318 University of Rome Tor Vergata, Rome, Italy; 6 Department of Biomedical and Neuromotor Sciences (DIBINEM), Alma Mater University of Bologna, Bologna, Italy; 7 Unit of Occupational Medicine, IRCCS Azienda Ospedaliero-Universitaria di Bologna, Bologna, Italy

**Keywords:** vestibular rehabilitation, vestibular physical therapy, Multiple Sclerosis, rehabilitation, neurorehabilitation, balance

## Abstract

Multiple Sclerosis (MS) is a chronic, inflammatory, and degenerative disease of the central nervous system. Balance impairments are very common symptoms among people with MS (PwMS). Vestibular physical therapy (VPT) is an evidence-based approach aimed at managing and improving gaze and balance stability, which could be used to mitigate balance disorders in MS. The objective of this systematic review with meta-analysis is to evaluate the effects of VPT in managing balance and dizziness in PwMS.

Electronic databases were searched in February 2025 (PubMed, Scopus and Cochrane) and screened by two blind reviewers. Risk of Bias was assessed using PEDro Scale. A meta-analysis was conducted to evaluate, across multiple studies, the effect sizes for two clinical outcomes: the Dizziness Handicap Inventory (DHI) and the Activities-specific Balance Confidence Scale (ABC).

After screening, 16 articles were included in the review, mostly comparing VPT with other conventional approaches. Analysis of the Risk of bias showed quality deficiencies in these studied with respect to the blinding of participants, assessor and physiotherapist. The meta-analysis showed significant efficacy of VPT against control using the DHI as outcome measure (Hedges’ g = 1.451, CI [0.190, 2.713]).

In conclusion, VPT is a safe approach in reducing dizziness-related disability of PwMS. Further studies are needed to refine assessment methods and explore individualized approaches.

## Introduction

Multiple Sclerosis (MS) is a chronic, inflammatory, and degenerative disease of the central nervous system, affecting approximately 2.8 million people globally.^1,2^ The signs and symptoms experienced by people with MS (PwMS) are highly variable, depending largely on the location of the lesions within the central nervous system. Poor dynamic balance, such as poor walking stability, is a hallmark of MS. With respect to the local trunk stability, even PwMS with no clinical evidence of gait impairment may experience balance alterations that, albeit not clinically evident, can be detected by analyzing the local trunk stability during walking.^3^ Balance impairments are evident in almost 86% of individuals with MS, and a certain fraction of these disorders could be related to vestibular system dysfunction.^4^ Moreover, one of the most common symptoms reported by PwMS is fatigue, to the extent that specific assessment scales have been developed to measure the perceived fatigue.^5^ Furthermore, heightened perception of fatigue is directly associated with lower quality of life.^5^

Vestibular physical therapy (VPT) is an evidence-based approach aimed at managing and improving gaze and balance stability through specific exercises designed to promote central compensation also in people with neurological disorders.^6,7^ VPT specifically targets neuroplastic mechanisms through structured, exercise-based protocols^7^ but the broader implementation of VPT is challenged by the perceived lack of foundational training in vestibular assessment and rehabilitation among healthcare professionals in neurorehabilitation pointed out by a recent survey.^8^ This gap in knowledge and skills may limit the availability and accessibility of VPT to patients who could benefit from it. While previous systematic reviews^6,9–11^ supported the effectiveness of VPT in managing balance impairments and dizziness in PwMS, they have been limited by a lack of updated evidence. Indeed, recent interventional studies have introduced novel VPT protocols,^12,13^ integrated assessments, and multimodal approaches that were not reported in earlier reviews of the literature. Furthermore, the clinical impact of VPT on specific validated outcome measures requires further quantification to guide individualized rehabilitative programs.

In light of this, this study has the main objective of providing a comprehensive update of the available literature incorporating recent advances in VPT by quantifying its clinical impact through a meta-analysis of validated outcome measures, namely, the Dizziness Handicap Inventory (DHI) and the Activities-specific Balance Confidence (ABC) scale.^14,15^ It is also important to remark that the review focuses exclusively on studies investigating VPT, without including general balance exercise programs that do not specifically target the vestibular system.

## Methods

This systematic review was performed according to the PRISMA (Preferred Reporting Items for Systematic Reviews and Meta-Analyses) statement^16^ and following the Cochrane Handbook for Systematic Reviews of Interventions.^17^ The protocol was regularly approved and published in an international prospective register of systematic reviews (PROSPERO CRD420251046971).

### PICOS question, search strategy and eligibility criteria

The research questions were formulated according to the PICOS strategy. The PICOS framework was applied to structure and systematically address the research question guiding this review: What is the role of VPT in improving balance and gait in PwMS?

Electronic databases searched in February 2025 were PubMed, Scopus and Cochrane. Search terms used were (“vestibular rehabilitation” OR “vestibular therapy” OR “vestibular training” OR “vestibular exercises”) AND (“multiple sclerosis” OR “MS” OR Multiple Sclerosis [MeSH]). Search terms were modified for each database and appropriate subheadings were used for each database searched (for details, see appendix A).

We searched all the available full texts written in English and published from inception to 18/02/2025, by considering the following inclusion criteria, under their respective PICOS components:

P (Population): Studies involving adult patients with a confirmed diagnosis of Multiple Sclerosis according to the diagnostic criteria used during the years of the study’s implementation.
I (Intervention): Studies focused on Vestibular Physical Therapy (VPT)
C (Comparison): Studies that included a comparison group (e.g., conventional therapy, other physical exercises, usual care, or no intervention).
O (Outcome): Studies that assessed balance, gait, dizziness, or related functional outcomes (e.g., DHI, ABC, gait parameters).
S (Study Design): All studies with randomized controlled trial designs were included. Case reports, conference papers and study protocols were not included in our study.

Exclusion criteria involved studies on people with other diseases known to be the primary cause of vestibular or balance dysfunction (e.g., stroke, traumatic brain injury, confirmed Meniere’s disease, Parkinson’s disease), and studies focused on other rehabilitative strategies that do not include a specific vestibular component.

### Study selection and data collection process

All studies were uploaded on an online dataset (RYYAN).^18^ Screening and data extraction were performed by two blinded reviewers (DP and GP) and, in case of disagreement, a third reviewer (II) was used to make a decision.

The first selection of studies was based on the title and abstract and, finally, full-text articles were examined. Study design and methodology were extracted from each study by two reviewers (DP and GP) and recorded on an Excel spreadsheet. The file reported the first author’s name and year of publication, the study design, the type of randomization where possible, the patient group features, the setting, the intervention in detail with the number and duration of sessions, the control group treatment, all the outcomes considered, and the study’s conclusions. All these procedures were implemented according to the PRISMA statement.^16^ Prisma checklist is available in the appendix B of the supplemental material.

### Risk of bias assessment

The aim of this review was to evaluate the efficacy of VPT in improving balance and dizziness symptoms in PwMS. Given that most of the included studies were randomized controlled trials, we selected the Physiotherapy Evidence Database (PEDro) Scale^19,20^ as a tool to assess the risk of bias for each study. This scale is a validated 11-item tool specifically designed to assess the methodological quality of physical therapy clinical trials. It assigns a score based on criteria such as eligibility, randomization, concealed allocation, and baseline comparability. Crucially, it evaluates the blinding of participants, therapists, and assessors, which was identified as a frequent source of bias in the included studies. The scale also accounts for adequate use of appropriate statistical analysis.

### Procedure of meta-analysis

The meta-analysis synthesized effect sizes across multiple studies for two clinical outcome measures commonly used to assess balance and dizziness-related disability: the DHI^14^ and the ABC.^15^ The DHI is a self-reported questionnaire that evaluates the perceived impact of dizziness on daily functioning, covering physical, emotional, and functional domains. The ABC scale, on the other hand, measures an individual’s confidence in maintaining balance while performing various daily activities. Only studies that included either the DHI or the ABC as outcome measures were considered eligible for inclusion. Specifically, we considered five articles for the DHI^12,21–24^ and three for the ABC.^22–24^ For each study, relevant data as group means, standard deviations, and sample sizes were individually extracted from the original publications.

The standardized mean difference (SMD) was calculated using Hedges’ g, which provides a correction for small sample bias. For the ABC scale, where higher scores indicate greater balance confidence, Hedges’ g was computed using the conventional formulation. For the DHI, where lower scores indicate a reduction in dizziness-related disability, the effect size was oriented so that positive values represent clinical improvement. Accordingly, the standardized mean difference for the DHI was calculated by subtracting the treatment mean from the control mean. This convention ensures a consistent interpretation across outcome measures, such that for both the DHI and ABC scales, positive Hedges’ g values indicate results favoring VPT.

A random-effects meta-analysis was performed using the DerSimonian–Laird method to account for between-study heterogeneity. Heterogeneity was assessed using Cochran’s Q statistic, defined as and quantified using the I^2^, which describes the percentage of total variation across studies due to heterogeneity rather than chance (Low Heterogeneity: 0%–40%; Moderate Heterogeneity: 30%–60%; Substantial Heterogeneity: 50%–90%; Considerable Heterogeneity: 75%–100%).^17^

Forest plots were generated to visualize the effect sizes and confidence intervals of individual studies, along with the pooled estimate ( $\bar{g}$ ). Study weights were represented by the size of the markers, scaled according to the inverse of the study variance compared to the total variance (within- and between-study).

To assess potential publication bias, a funnel plot was generated by plotting the standard error of each study against its estimated effect size.

To complement the visual inspection, Egger’s regression test was performed to statistically evaluate the degree of funnel plot asymmetry, with a significant result indicating a potential small effect or bias.

The standardized effect size was modeled using linear regression as follows:

$$\frac{g_{i}}{SE_{i}}=\beta_{0}+\beta_{1}\cdot \;\frac{1}{SE_{i}}+\varepsilon_{i}$$

where *g*_*i*_ is the effect size (Hedge’s *g*) of i-th study, *SE*_*i*_ the standard error of the study, *ε*_*i*_ the error term, and β_0_ and β_1_ are estimated intercept and slope of the linear regression model, respectively.

For the meta-analysis, we considered the VPT as a treatment condition and “no intervention” or “usual care” as control condition. However, for one study^24^ the control condition was cardiovascular endurance training, while the treatment consisted of VPT combined with cardiovascular endurance training. Therefore, we considered cardiovascular endurance training as the baseline condition, with VPT as an add-on treatment.

All analyses were conducted using custom scripts written in Python (version 3.11) within the Google Colaboratory (Colab) environment, utilizing core libraries for statistical computations.

## Results

### Study selection

Initially, 170 articles were identified. After removing 61 duplicates, 109 articles were screened for their title, resulting in the exclusion of other 66 articles. Subsequently, the abstracts of the 43 remaining articles were reviewed, leading to the further exclusion of 15 articles (14 registered protocols, 1 conference paper). Finally, the 28 articles that passed the abstract screening underwent full-text review. During this stage, 12 articles were excluded (2 due to being in a non-English language, 3 due to full-text unavailability and the others for not complying with the inclusion criteria). In the end, 16 articles were included in the final dataset. The PRISMA flow chart illustrating the screening process is shown in Figure 1.^16^

**Figure 1.**
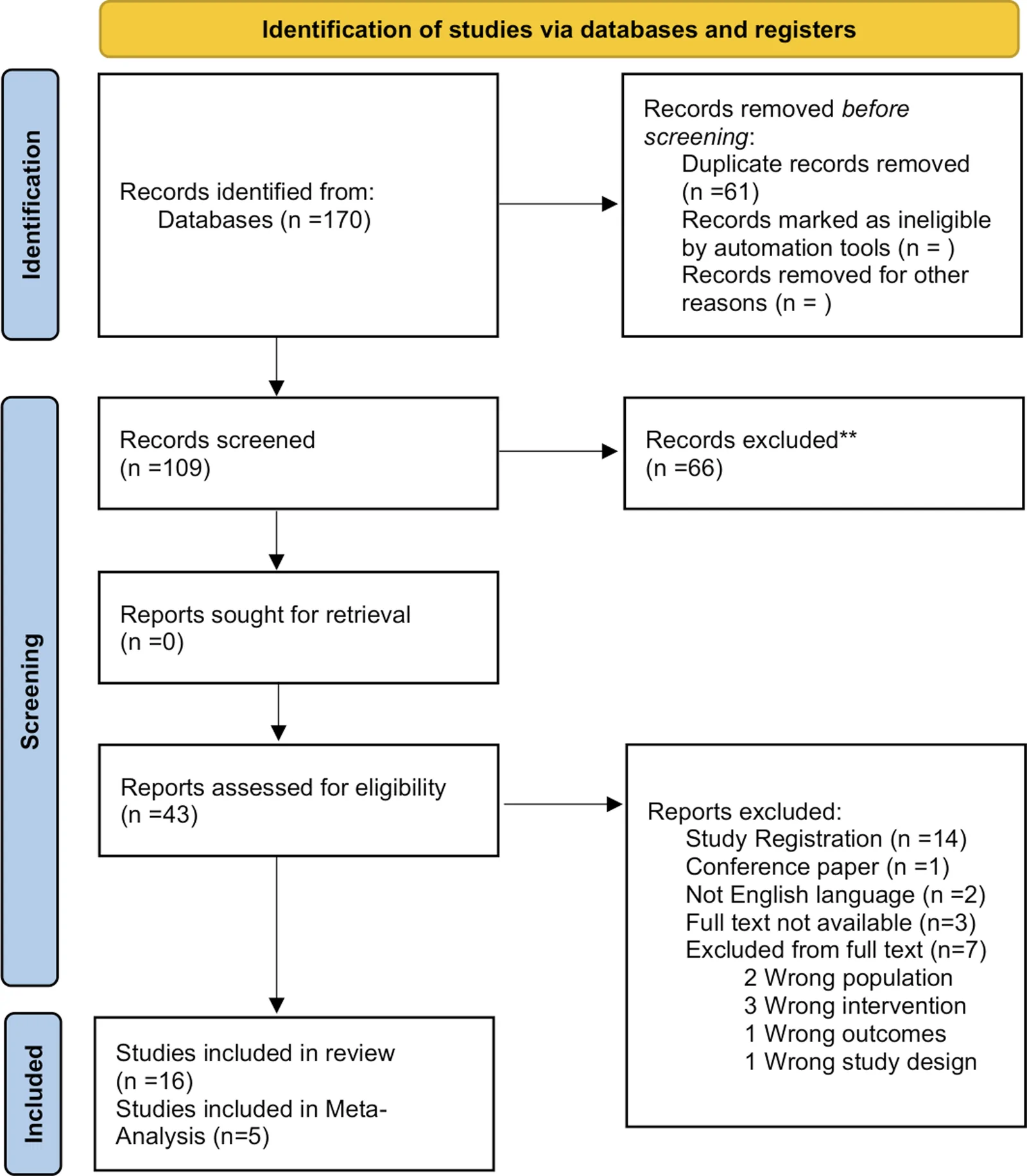
flow chart summarizing the article screening process. After applying the inclusion and exclusion criteria, 16 articles were finalized for inclusion in the review.

### Results of the included studies

The studies included in this review are interventional in character, investigating the efficacy of VPT using various comparative designs. Several of them compared directly VPT to traditional rehabilitation treatments, such as muscle strengthening exercises or general balance exercises.^22,25–28^ Other studies, instead, used more complex comparative frameworks. For example, Afrasiabifar et colleagues^29^ compared outcomes between groups that received Cawthorne-Cooksey exercises, Frenkel exercises, or routine care, whereas Lotfi et colleagues^23^ compared VPT to noisy galvanic vestibular stimulation and a no-intervention control group. Additional comparisons included VPT against aerobic exercise^30^ and stretching or bicycle ergometry versus no intervention.^13,21^ Furthermore, another study^24^ investigated the combined effect of VPT and cardiovascular endurance training against endurance training alone. Finally, we found growing interest in multimodal therapy approaches,^12^ which combined VPT with intermittent theta burst stimulation (iTBS). Complete results are summarized in Table 1.

**Table 1.** The table summarizes the main features of the study included in the review. Abbreviations: RCT: randomized controlled trial; MFIS: modified fatigue impact scale; DHI: dizziness handicap inventory; BDI: Beck Depression Inventory; SOT: sensory organization test (posturography); 6MWT: 6-minute walk test; BBS: Berg balance scale; ABC: activities-specific balance confidence scale; TBG: Tinetti Balance and Gait scale; EDSS: expanded disability status scale; CDP-SOT: computerized dynamic posturography – sensory organization test; FSS: fatigue severity scale; 2MWT: 2-minute walk test; T25FW: timed 25-foot walk. “/” was used if more groups were used as comparison.

Authors, year	Participants	Intervention	Comparison	Outcome	Conclusion
Hebert, 2011	38	VPT (Upright postural control and eye movement exercises)	Bicycle ergometry, stretching, Usual care	MFISSOTDHIBDI-II6MWT	The VPT group had greater improve- ments in fatigue, balance, and disability due to dizziness or disequilibrium compared with the exercise control group and usual care control group.
Study design: RCT					
	Average age: VPT: 46.8 ± 10.5 Bicycle ergometry: 42.6 ± 10.4 Usual care: 50.2 ± 9.2				
	
Ozgen, 2016	40	VPT (gaze and postural stability) + ambulation exercise	Usual care	Romberg Test	VPT is a low-cost, simple, and effective treatment method for improving balance, quality of life, and psychology in MS patients.
Study design: RCT	Average age: VPT: 42.5 Usual care: 39.5			Tandem Romberg Test	
				Foam Romberg Test	
				Static Posturography	
				6MWT	
				Five Times Sit-to-Stand Test BBS	
				Timed Up and Go	
				Functional Gait Assessment	
				Dynamic Gait IndexABC	
				DHI	
				Multiple Sclerosis Quality of Life-54	
				BDI	
Karami, 2018 Study design: RCT	72 Average age: Overall: 32.7 ± 7.4	Cawthorne and Cooksey exercise	Frenkel exercise/Usual care	Fatigue Impact Scale	Vestibular rehabilitation exercise is more effective than the Frenkel exercise in reducing fatigue.
Shady, 2018Study design: RCT	30 Average age: VPT: 30.7 ± 8.5 Conventional therapy: 29.1 ± 9.1	VPT (gaze stability and reduction of postural unsteadiness, improving gait stability)	Conventional therapy (muscle strengthening, postural reeducation)	EDSS MFIS Vertigo Symptom Scale Biodex Balance System Overall Stability Index Anterior-Posterior Stability Index Medio-Lateral Stability Index	VPT has positively improved the stability functions in the patients with relapsing-remitting MS.
Hebert, 2018 Study design: RCT	88 Average age: VPT: 46.5 ± 8.8 Usual care: 43.0 ± 10.8	BEEMS (balance and eye-movement exercises)	Usual care	CDP-SOT DHI MFIS Short Form-36 Health Survey	Participants with brainstem/cerebellar lesions showed greater improvements at 6 weeks than those without lesions for both CDP-SOT and MFIS total and physical subscale scores. However, differences were small and not sustained at 14 weeks. Furthermore, there was no difference based on brainstem/cerebellar involvement for DHI.
Tramontano, 2018 Study design: RCT	30 Average age: VPT: 50.6 ± 11.7 Conventional therapy: 45.8 ± 10.9	VPT (gaze stability and postural stability in a standing position on a foam cushion).	Conventional therapy	EDSS Barthel Index TBG BBS FSS 2MWT T25FW	VPT might have positive effects in MS hospitalized patients on balance and gait functions, on global activity and on the perception of the fatigue.
Tramontano, 2020Study design: RCT	16Average age: iTBS: 51.9 ± 3.8 Sham: 51.6 ± 10.9	iTBS + VPT	sham iTBS + VPT	TBG BBS Barthel Index FSS 2MWT T25FW	c-iTBS combined with VPT improves gait and fatigue.
Lotfi, 2021 Study design: RCT	24 Average age: VPT: 43.2 ± 6.5 Galvanic: 44.2 ± 9.8 Usual care: 38.1 ± 7.9	VPT (eye-head coordination, sitting, static and dynamic standing, and walking)	Noisy Galvanic Vestibular Stimulation/Usual care	SOT DHI ABC	VPT is effective in reducing dizziness and improving balance in PwMS.
Loyd, 2022 Study design: RCT	41 Average age: VPT: 56.1 ± 10.0 Cardiovascular: 50.0 ± 13.1	VPT (gaze stability training and postural stability training, with an additional ∼45 min of lower extremity strengthening and cardiovascular endurance training)	Cardiovascular endurance training	ABC DHI computerized Dynamic Visual Acuity Functional Gait Assessment	VPT was found to be statistically equivalent to a strength and aerobic exercise intervention at improving dizziness and balance.
Mohamed, 2024 Study design: RCT	30 Average age: VPT: 40.3 ± 4.2 conventional therapy: 42.5 ± 6.7	Conventional + VPT (Cawthorne Cooksey exercises)	Conventional vestibular therapy	Trunk kinetic and kinematic	Cawthorne Cooksey exercises result in improvement in trunk movements and muscle power.
Darwish, 2025 Study design: RCT	40 Average age: VPT: 46.7 ± 12.4 Aerobic: 41.8 ± 11.8	VPT (aerobic exercise + designed vestibular rehabilitation)	Aerobic exercise	Oral Symbol Digit Modalities Test California Verbal Learning Test-II Brief Visuospatial Retention Test–Revised	VPT enhances many cognitive functions in patients with relapsing-remitting MS.
Uzunoğlu, 2025	27	VPT (vestibular exercise)	Conventional therapy (kinetic and kinematic parameters of gait)	Gait analysis 2MWT 10-Meter Walk Test Single-Leg Standing Test Five Times Sit-to-Stand Test Visual Acuity Test	Integrating VPT with conventional training is expected to enhance the effectiveness of exercise interventions on kinetic and kinematic gait parameters.
Study design: RCT	Average age: VPT: 44.4 ± 3.6 Conventional therapy: 45.9±2.7				
	

### Risk of bias assessment

The risk of bias was assessed using the PEDro Scale, a commonly employed tool to evaluate the methodological quality of randomized controlled trials. The majority of studies were characterized as moderate to high-quality, with the most frequent sources of bias being related to the lack of blinding of participants, therapists, and assessors (Figure 2).

**Figure 2.**
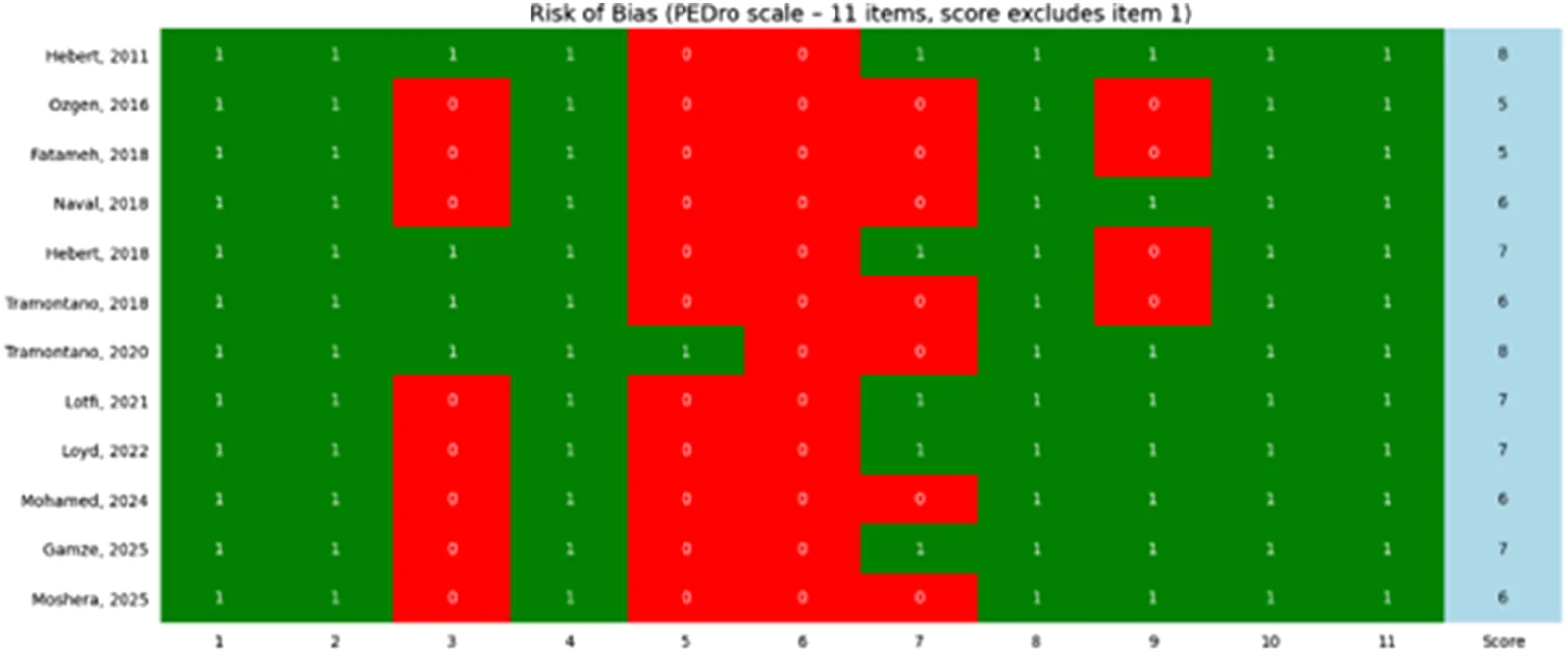
Risk of bias of studies included in the review.

### Results of meta-analysis

A random-effects meta-analysis was carried out on five studies assessing the impact of interventions on the DHI. The pooled effect size, calculated using Hedges’ g with a preliminary sign inversion to ensure consistency with the principle that lower DHI scores correspond to greater stability, was 1.451 with a 95% confidence interval of [0.190, 2.713] (Table S2, available as Supplemenatry Material), indicating a statistically significant improvement in DHI scores favoring the treatment groups (Figure 3).

**Figure 3.**
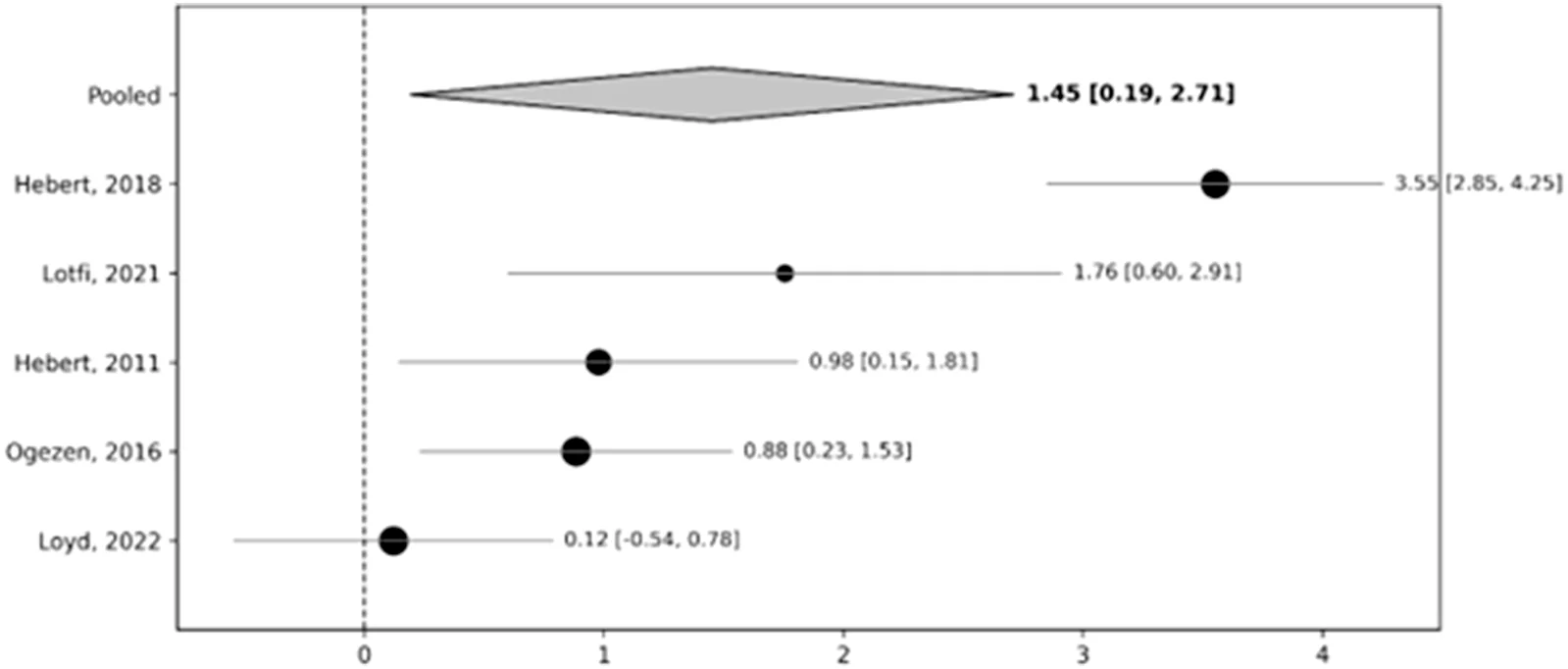
Results of meta-analysis (DHI). Forest plot showing the effect sizes (with 95% confidence intervals) from individual studies and the pooled estimate from meta-analysis. Studies are sorted in ascending order of effect size (g). The size of each dot reflects the relative weight of the study in the random-effects model, where the weight ( ω_(i)) is defined as the inverse of the variance of Hedge’s g for each study.

Substantial heterogeneity was observed among the studies (Q = 54.42, df = 4, I^2^ = 92.6%), suggesting considerable variability in effect sizes beyond what would be expected by chance.

Visual inspection of the funnel plot did not reveal substantial asymmetry overall. The individual study estimates appeared to be symmetrically distributed around the pooled effect size, indicating no clear pattern of systematic publication bias. Specifically, studies with smaller samples did not report disproportionately larger effect sizes, which is a common indicator of small-study effects.

However, it is worth noting that two of the most precise studies, located in the upper part of the funnel for their low standard errors, fell substantially outside the expected funnel boundaries. Interestingly, these deviations occurred in opposite directions, effectively balancing each other and limiting their influence on the asymmetry statistic. One of these studies, in particular, showed a markedly high effect size despite reporting group means comparable to the other studies; this discrepancy is likely due to its larger sample size, which substantially reduced the standard error and thus magnified the standardized effect estimate.

The Egger’s regression test was, then, performed to statistically assess asymmetry. The intercept of the regression line was 2.33, with a p-value of 0.832, indicating no statistically significant deviation from symmetry. Nonetheless, given the small number of included studies and the presence of high-precision outliers, caution is warranted in interpreting both the visual and statistical indicators of publication bias (see Figure 4).

**Figure 4.**
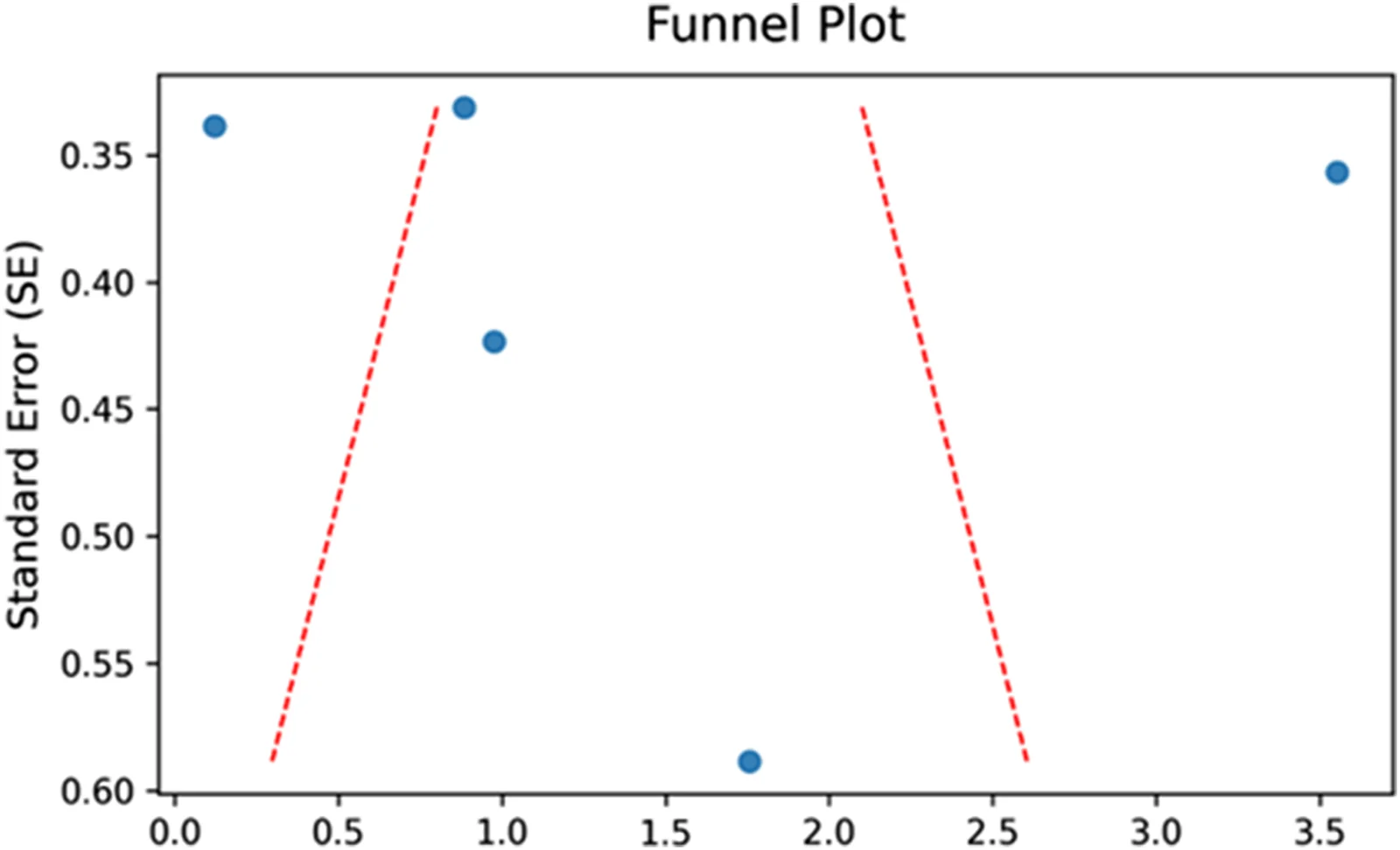
Funnel plot shows the results of meta-analysis on DHI. Points inside the funnel show consistency between studies regarding the proportion between effect size and variance of the effect size. The *x*-axis represents the effect size measured using Hedge’s g.

These findings suggest that, across the included studies, interventions were associated with a meaningful and statistically significant reduction in DHI scores, though the high heterogeneity pointed out by the Cochran’s Q statistics warrants cautious interpretation.

With respect to the ABC scale, the meta-analysis was performed on three studies evaluating the effect of interventions on this scale. The pooled effect size (Hedges’ g) was 0.790, with a 95% confidence interval of [-0.315, 1.895] (Table S3, available as Supplementary Material), indicating a non-significant overall effect (Figure 5).

**Figure 5.**
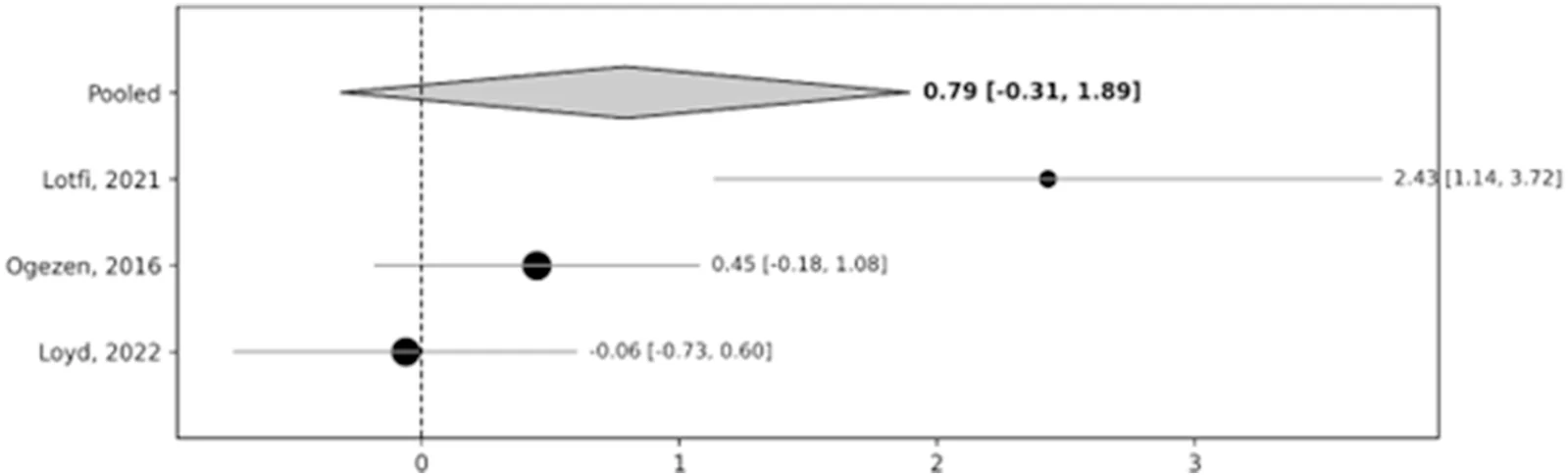
Results of meta-analysis (ABC). Forest plot displaying individual study effect sizes and the pooled estimate with 95% confidence intervals. Studies are sorted in ascending order of effect size (g). The size of each dot reflects the relative weight of the study in the random-effects model.

Substantial heterogeneity was observed among studies, as indicated by the Cochran’s Q = 11.32 (df = 2) and the I^2^ statistic of 82.3%. This suggests that a large proportion of the variability in the effect sizes may reflect genuine differences among studies rather than random error.

Regarding the potential publication bias, visual inspection of the funnel plot did not reveal notable asymmetry. Egger’s regression test further supported this observation, with a non-significant intercept of 6.49 (*p* = 0.263), indicating no statistical evidence of small-study effects or selective reporting.

However, the number of included studies was limited, which reduces the reliability and statistical power of both the funnel plot and Egger’s test in detecting publication bias (Figure 6).

**Figure 6.**
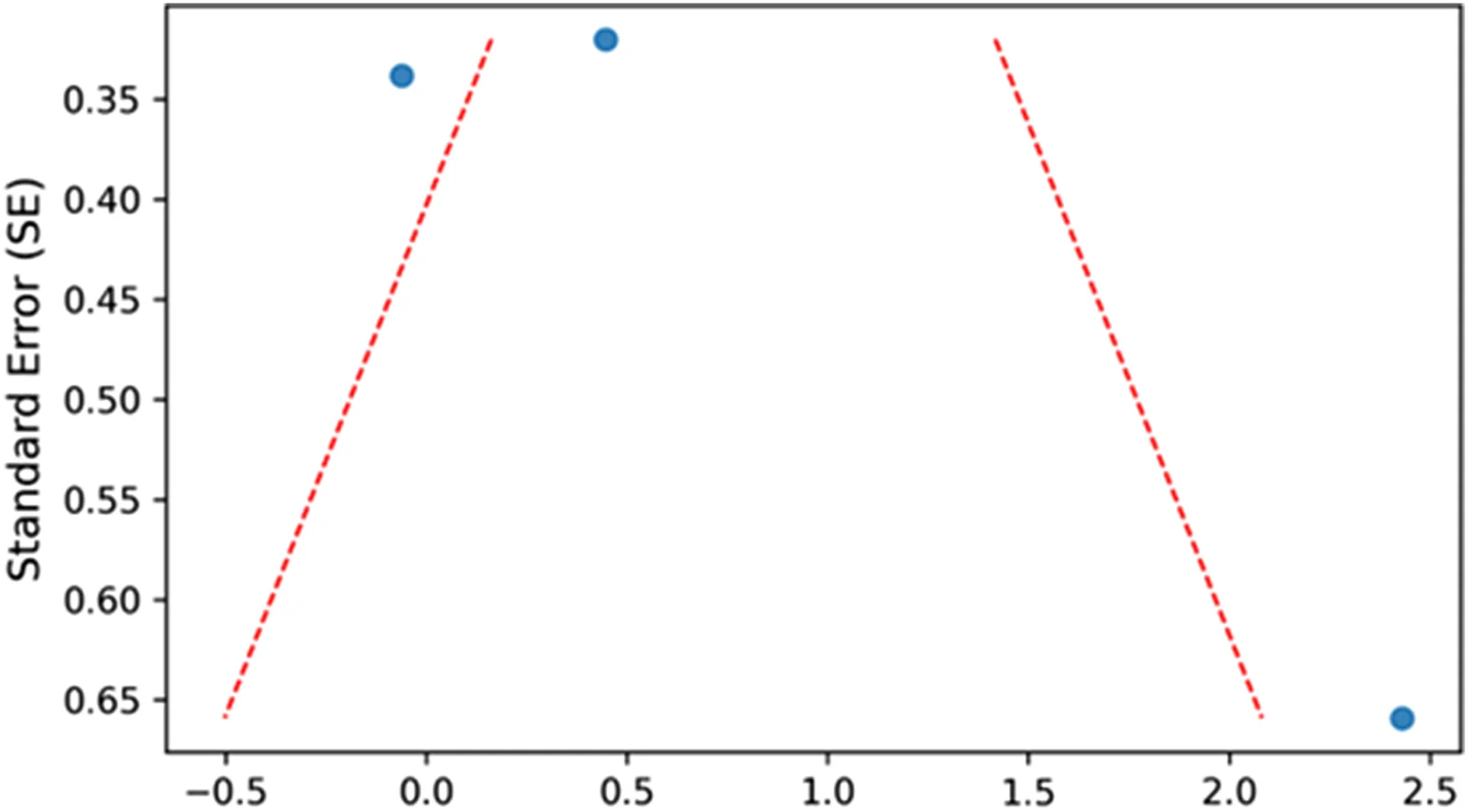
Funnel plot shows the results of meta-analysis on ABC. The *x*-axis represents the effect size measured using Hedge’s g.

Overall, while the pooled estimate suggested a trend toward improved ABC scores, the result did not reach statistical significance and is accompanied by substantial heterogeneity.

## Discussion

This systematic review aimed to evaluate the effects of VPT in managing balance and dizziness in PwMS. Previous reviews have already reported the effectiveness of VPT in improving balance and reducing the related symptoms, such as dizziness and fatigue, in neurological patients and in PwMS.^6,9–11^ In this context, however, we believe it was necessary not only reviewing the evidence about the clinical efficacy of VPT but also quantifying the extent to which common outcome measures in clinical trials assess the efficacy of VPT and how such interventions could be tailored to individual patients.

Both the meta-analyses conducted in this study, the first on studies using the DHI as outcome measure and the second on studies using the ABC, showed high levels of heterogeneity, indicating significant variability in the effect estimates across included trials. Thus, variations in study design, sample characteristics, types of intervention, or outcome assessment methodologies could be influencing the results. This result is in accordance with a previous review article,^11^ which reported significant variability in VPT interventions among PwMS. Our study confirms this trend, demonstrating further that even with the inclusion of a larger number of recent trials, the high level of heterogeneity persists.

Given the nature of MS, which is marked by substantial inter-individual variability in symptom presentation, disease progression, and functional status, the effort of reducing heterogeneity may be inherently challenging. Indeed, this intrinsic variability across PwMS may limit the ability to achieve homogeneity, even with standardized protocols. Such heterogeneity diminishes the confidence in a single pooled estimate and emphasizes the importance of interpreting results with caution. Beyond the intrinsic characteristics of the sample, the observed heterogeneity is significantly increased by the substantial differences between the VPT interventions themselves. The included studies utilize a broad range of protocols, from traditional exercises, such as the Cawthorne-Cooksey approach, to more dynamic and complex multisensory integration exercises. This spectrum of complexity, moving from simple compensatory movements to integrated dynamic balance training, inevitably impacts the magnitude of clinical outcomes and contributes to the large variance in effect sizes.

Despite this substantial heterogeneity and the need for cautious interpretation of pooled estimates, the overall qualitative synthesis reveals a consistent trend supporting the clinical utility of VPT in PwMS. Our present review identified a remarkable body of interventional studies providing preliminary evidence in support of the efficacy of VPT. Studies consistently report improvements in postural stability, fatigue reduction, enhancement of gait parameters, and in some cases, cognitive benefits.^12,13,21–30^ These outcomes may be partly attributed to the flexibility of VPT protocols, which allow clinicians to incorporate a variety of exercises based on individual patient needs. However, despite this potential for personalization, most studies lack detailed information regarding the specific assessment tools used to evaluate patients’ needs, the precise VPT protocols implemented, and how these protocols are tailored to the individual cases. This limited reporting hinders the development of clear guidelines and highlights the need for structured and flexible evaluation frameworks to support individualized treatment planning. Even considering the outcomes of meta-analyses reported in previous review articles,^11^ a significant lack of instrumental measurements in the methodologies of the included RCTs remains evident. The absence of objective postural stability assessments, such as computerized posturography or wearable sensors, represents a missed opportunity to gain deeper insights into balance assessment.

One implication of the results of our review study is that vestibular-specific evaluations, largely underestimated in MS rehabilitation, should be integrated more systematically to customize the rehabilitation plan.^4,31,32^ Although these instrumental measurements may not always serve as responsive outcome measures because of the potential effects of central compensation, they are essential for establishing a comprehensive clinical baseline. Furthermore, such objective data are crucial for guiding clinical decision-making, allowing for the selection of more targeted and effective therapeutic strategies tailored to the specific vestibular profile of the patient. Recent evidence^4^ indicates that vestibular dysfunction is prevalent even in PwMS, suggesting that clinicians may be overlooking treatable balance disorders. A comprehensive vestibular assessment battery that includes both instrumented and clinical tests, such as the video Head Impulse Test (vHIT),^4,31^ vestibular-evoked myogenic potentials (VEMPs),^32^ and functional challenges may support more precise identification of vestibular deficits and guide tailored interventions. Given the high prevalence of vestibular dysfunction in pwMS, studying these aspects is essential for the personalized therapeutic programs. For example, identifying specific VOR deficits through vHIT may allow clinicians to tailor treatment protocols by prioritizing gaze stabilization exercises for patients with documented vestibular hypofunction. Conversely, patients showing impaired postural control, but preserved VOR, might benefit more from dynamic multisensory integration exercises.

Moreover, without specific and objective assessments of vestibular function, it remains unclear whether improvements following VPT are due to targeted effects on the vestibular system or rather to general enhancements in sensorimotor integration.

Incorporating such evaluations in future studies would not only clarify the mechanisms underlying VPT efficacy but also strengthen the rationale for its clinical use by demonstrating its direct impact on vestibular function. For instance, evaluating the characteristics of compensatory saccades through vHIT could help determine whether VPT leads to improved gaze stability.^33^ This, in turn, would provide insight on the physiological basis of observed clinical improvements and support the identification of specific subgroups of patients, who can more likely benefit from this type of training.

Moreover, instrumental assessments, such as gait analysis and wearable inertial sensors, could be utilized to evaluate patients in ecological, real-world settings, thereby enhancing the clinical relevance of the data collected.^34^ These tools also offer high sensitivity to subtle postural and gait impairments, enable continuous, real-time feedback, and support the precise tailoring and adjustment of VPT interventions to individual patient needs.^35^ Their integration into clinical pathways can enable more refined progress monitoring and dynamic adjustment of customized rehabilitation programs.^34^ Furthermore, these specific assessments allow clinicians to precisely tailor rehabilitation programs that also manage fatigue while optimizing gait parameters and maintaining or increasing walking endurance.^36^ Given the direct relationship between the vestibular system, autonomic regulation, and cognitive functions,^37^ it is crucial to incorporate these dimensions into the personalization of treatment protocols. There is a clear need for a greater number of RCTs that include these specific outcome measures. Such research would be essential to verify whether VPT approach can significantly improve cross-modal aspects not directly related to balance, such as cognitive processing speed or spatial memory.

A crucial aspect for the clinical implementation of VPT in PwMS is the management of perceived fatigue. Personalized treatment must carefully consider the patient’s fatigue levels to determine the appropriate “dosage” of exercise. The intensity and duration of vestibular exercises should be tailored to the individual’s daily energy threshold. For instance, in people with high levels of fatigue, shorter but more frequent sessions might be preferable to long, exhaustive protocols.

It is also critical to identify which subgroups of PwMS are most likely to benefit from VPT, also by considering that vestibular reflex and compensatory oculomotor functions deficits are associated with a greater MS-related disability.^31^ In future research, it would also be valuable to include fatigue as a demographic and clinical variable, as its severity and impact may influence treatment responsiveness and help to further characterize the efficacy of VPT across different PwMS subgroups. In light of these considerations, it will be crucial to conduct future meta-analyses by subdividing patients into subgroups. This implies that clear reporting of all demographic and clinical parameters in future clinical trials is essential.

Future research should also explore more in depth how to enhance the outcomes of VPT by combining it with other types of treatment, such neuromodulatory techniques.^12^ This combined approach, which is gaining increasing attention in neurorehabilitation,^38,39^ may offer synergistic effects by priming cortical circuits.

### Potential limitations of the study

One potential limitation of the study concerns the limited number of studies we could include in the meta-analysis, which prevented us the meta-analysis on separate patient subgroups. Future research should investigate more in depth the potential influential factors that emerged from our qualitative synthesis. For instance, the disease course and MS subtype are likely to influence the central nervous system’s adaptive capacity yielding to vestibular compensation. Furthermore, patient age and baseline disability levels should be analyzed as predictors of treatment response.

However, based on the rationale of VPT, we can hypothesize that patients with low to moderate disability might benefit more from VPT. Furthermore, it should be noted that the diagnostic criteria for MS have been modified over the years. Although to ensure methodological correctness, the gold standard parameters at the time of the study’s realization were considered, this detail could contribute to the heterogeneity among studies. Another limitation may relate to the search strategy we adopted, as it focused on broad clinical intervention terms which might have excluded studies centered on the underlying mechanisms of the VPT (e.g., vestibular adaptation, compensation, or habituation) but that did not include our search keywords. This choice, while intentional to target interventional trials, may have restricted the total body of evidence retrieved.

## Conclusion

VPT is a safe approach that demonstrates significant efficacy in reducing dizziness-related disability among PwMS. While a positive trend was observed for balance-related confidence, the results were non-significant and characterized by high heterogeneity. While the available evidence supports its clinical utility, further research is needed to refine assessment methods and explore individualized approaches. Incorporating appropriate evaluation tools could be essential to personalize care, optimize outcomes, and advance the clinical implementation of VPT in the rehabilitation of PwMS.

## Supplemental material

Suppplemental Material - Effectiveness of vestibular physical therapy on balance and dizziness in people with multiple sclerosis: A systematic review and meta-analysis Suppplemental Material for Effectiveness of vestibular physical therapy on balance and dizziness in people with multiple sclerosis: A systematic review and meta-analysis by Diego Piatti, Gianluca Paolocci, Daniel Hector Verdecchia3, Maria Grazia Grasso, Gianfranco Bosco, Iole Indovina, Marco Tramontano in Journal of Vestibular Research

Suppplemental Material - Effectiveness of vestibular physical therapy on balance and dizziness in people with multiple sclerosis: A systematic review and meta-analysis Suppplemental Material for Effectiveness of vestibular physical therapy on balance and dizziness in people with multiple sclerosis: A systematic review and meta-analysis by Diego Piatti, Gianluca Paolocci, Daniel Hector Verdecchia3, Maria Grazia Grasso, Gianfranco Bosco, Iole Indovina, Marco Tramontano in Journal of Vestibular Research

Suppplemental Material - Effectiveness of vestibular physical therapy on balance and dizziness in people with multiple sclerosis: A systematic review and meta-analysis Suppplemental Material for Effectiveness of vestibular physical therapy on balance and dizziness in people with multiple sclerosis: A systematic review and meta-analysis by Diego Piatti, Gianluca Paolocci, Daniel Hector Verdecchia3, Maria Grazia Grasso, Gianfranco Bosco, Iole Indovina, Marco Tramontano in Journal of Vestibular Research

Suppplemental Material - Effectiveness of vestibular physical therapy on balance and dizziness in people with multiple sclerosis: A systematic review and meta-analysis Suppplemental Material for Effectiveness of vestibular physical therapy on balance and dizziness in people with multiple sclerosis: A systematic review and meta-analysis by Diego Piatti, Gianluca Paolocci, Daniel Hector Verdecchia3, Maria Grazia Grasso, Gianfranco Bosco, Iole Indovina, Marco Tramontano in Journal of Vestibular Research
