# Supplementary material for: Effectiveness of vestibular physical therapy on balance and dizziness in people with multiple sclerosis: A systematic review and meta-analysis
Source: J Vestib Res. 2026 May 5;36(6):378–91. doi: 10.1177/09574271261444952 (PMC13672864; doi:10.1177/09574271261444952)
Supplement: Supplementary file 1 [file sj-pdf-1-ver-10.1177_09574271261444952.pdf]

Search terms were modified for each database and appropriate subheadings were used for each database searched. The following strings were used in the respective databases:

Pubmed: ("vestibular rehabilitation" OR "vestibular therapy" OR "vestibular training" OR "vestibular exercises")
AND ("multiple sclerosis" OR "MS" OR Multiple Sclerosis[MeSH])

Scopus: TITLE-ABS-KEY ("vestibular rehabilitation" OR "vestibular therapy" OR "vestibular training" OR "vestibular exercises") AND ("multiple sclerosis" OR "MS")

Cochrane: ("vestibular rehabilitation" OR "vestibular therapy" OR "vestibular training" OR "vestibular exercises")
AND ("multiple sclerosis" OR "MS")
