# Supplementary material for: Effectiveness of vestibular physical therapy on balance and dizziness in people with multiple sclerosis: A systematic review and meta-analysis
Source: J Vestib Res. 2026 May 5;36(6):378–91. doi: 10.1177/09574271261444952 (PMC13672864; doi:10.1177/09574271261444952)
Supplement: Supplementary file 3 [file sj-pdf-3-ver-10.1177_09574271261444952.pdf]

Table S2

Study	VPT group (N)	DHI (Mean ± SD)	Control group (N)	DHI (Mean ± SD)
Hebert, 2011	12	29.30 ± 18.60	13	44.80 ± 11.60
Ogezen, 2016	20	34.00 ± 10.70	20	46.00 ± 15.50
Hebert, 2018	39	27.80 ± 3.80	42	40.70 ± 3.40
Lotfi, 2021	8	10.25 ± 12.42	8	33.75 ± 12.89
Loyd, 2022	18	32.37 ± 20.00	17	34.81 ± 19.30
	Results:	*Hedges' g = 1.451*	*95% CI [0.190, 2.713]*	*Q = 54.42, df = 4, I^2 = 92.6%*<br>*P-Value=0.02*

Table description: Data and results of meta-analysis on DHI, N indicates the number of participants within each group.

Table S3

Study	ABC group	ABC (Mean ± SD)	Control group	ABC (Mean ± SD)
Ogezen, 2016	20	54.06 ± 17.50	20	46.25 ± 16.70
Lotfi, 2021	8	91.81 ± 5.66	8	62.62 ± 15.03
Loyd, 2022	18	75.11 ± 17.00	17	76.20 ± 17.21
	Results:	*Hedges' g = 0.790*	*95% CI [-0.315, 1.895]*	*Q = 11.32, df = 2, I^2 = 82.3%*<br>*P-Value=0.16*

Table description: Data and results of meta-analysis on ABC scale.
