# Supplementary material for: Effectiveness of vestibular physical therapy on balance and dizziness in people with multiple sclerosis: A systematic review and meta-analysis
Source: J Vestib Res. 2026 May 5;36(6):378–91. doi: 10.1177/09574271261444952 (PMC13672864; doi:10.1177/09574271261444952)
Supplement: Supplementary file 4 [file sj-pdf-4-ver-10.1177_09574271261444952.pdf]

Supplementary Material

The following supplemental material accompanies the manuscript and is provided to aid transparency and reproducibility:

Tables

Tables and descriptions of the meta-analysis.

Appendix A – Search Strategy

Description: This supplemental file details the full electronic database search strategy used for the systematic review, including keywords, Boolean operators, and limits applied.

Appendix B – PRISMA Checklist

Description: This supplemental file contains the completed PRISMA 2020 checklist documenting adherence to reporting standards for systematic reviews.
